# Glucosylceramide Administration as a Vaccination Strategy in Mouse Models of Cryptococcosis

**DOI:** 10.1371/journal.pone.0153853

**Published:** 2016-04-15

**Authors:** Visesato Mor, Amir M. Farnoud, Ashutosh Singh, Antonella Rella, Hiromasa Tanno, Keiko Ishii, Kazuyoshi Kawakami, Toshiya Sato, Maurizio Del Poeta

**Affiliations:** 1 Department of Molecular Genetics and Microbiology, Stony Brook University, 145 Life Sciences Building, Stony Brook, NY, 11794, United States of America; 2 Department of Science of Nursing Practice, Tohoku University Graduate School of Medicine, 2–1 Seiryo-cho, Aoba-ku, Sendai, Miyagi, 980–8575, Japan; 3 Department of Medical Microbiology, Mycology and Immunology, Tohoku University Graduate School of Medicine, 2–1 Seiryo-cho, Aoba-ku, Sendai, Miyagi, 980–8575, Japan; 4 Kohjin Life Sciences Co., Ltd. 1–3 Yurakucho 1-Chome.Chiyoda-KU, Tokyo, 100–0006, Japan; 5 Veterans Administration Medical Center, Northport, NY, 11768, United States of America; University of Michigan Health System, UNITED STATES

## Abstract

*Cryptococcus neoformans* is an opportunistic fungal pathogen and the causative agent of the disease cryptococcosis. Cryptococcosis is initiated as a pulmonary infection and in conditions of immune deficiency disseminates to the blood stream and central nervous system, resulting in life-threatening meningoencephalitis. A number of studies have focused on the development of a vaccine against *Cryptococcus*, primarily utilizing protein-conjugated components of the *Cryptococcus* polysaccharide capsule as antigen. However, there is currently no vaccine against *Cryptococcus* in the clinic. Previous studies have shown that the glycosphingolipid, glucosylceramide (GlcCer), is a virulence factor in *C*. *neoformans* and antibodies against this lipid inhibit fungal growth and cell division. In the present study, we have investigated the possibility of using GlcCer as a therapeutic agent against *C*. *neoformans* infections in mouse models of cryptococcosis. GlcCer purified from a non-pathogenic fungus, *Candida utilis*, was administered intraperitoneally, prior to infecting mice with a lethal dose of *C*. *neoformans*. GlcCer administration prevented the dissemination of *C*. *neoformans* from the lungs to the brain and led to 60% mouse survival. GlcCer administration did not cause hepatic injury and elicited an anti-GlcCer antibody response, which was observed independent of the route of administration and the strains of mouse. Taken together, our results suggest that fungal GlcCer can protect mice against lethal doses of *C*. *neoformans* infection and can provide a viable vaccination strategy against *Cryptococcus*.

## Introduction

*Cryptococcus* is an opportunistic fungal pathogen and the most common cause of fungal meningitis. *Cryptococcus* infections, caused by *Cryptococcus neoformans* and *Cryptococcus gattii*, are initiated by inhaling fungal spores, which can survive and remain latent inside the alveolar macrophages. In conditions of immunosuppression, the fungus can disseminate from the lungs to the central nervous system, invade the brain, and cause fungal meningitis. Cryptococcal meningitis afflicts a large population worldwide leading to more than 600000 deaths per year [1].

In recent years, there has been increased attention on the development of vaccines against cryptococcosis and other fungal infections. A variety of vaccine formulations have been suggested against *Cryptococcus* infections. Glucuronoxylomannan (GXM), the main component of the polysaccharide capsule in *Cryptococcus*, has been used as a vaccine antigen in conjugation with a protein carrier (GXM-tetanus toxoid vaccine); however, this vaccine results in the generation of both protective and deleterious antibodies and does not provide a suitable candidate for translation to clinic [2–4]. Peptide mimotopes (mimotopes are mimetics that are able to elicit antibodies that bind the native antigen [5]) of the polysaccharide capsule, have also been suggested as potential vaccines [6, 7]; however, disadvantages include the important risk of unpredictable mimotope responses if the elicited antibodies are conformation-dependent [5]. Culture-based vaccine candidates against *Cryptococcus* infections have also been proposed in recent years, these include *C*. *neoformans* culture filtrate antigen [8] and protein preparations from *C*. *gattii* administered prior to infection [9]. Finally, genetically engineered *Cryptococcus* strains that generate cytokines [10, 11], and immunomodulatory glycolipids [12] have been recently proposed as vaccine candidates. Despite the number of vaccine candidates that have been proposed, currently no vaccines exist against cryptococcosis in the clinic and the search for suitable vaccines is still ongoing.

Evidence from the literature suggests that glycolipids might be an appropriate candidate for vaccine development against cryptococcosis. Our laboratory recently reported the characterization of a *Cryptococcus* mutant, Δ*sgl1*, which accumulated the glycolipid, sterylglycoside, and led to complete mice survival when applied as a vaccine prior to infection. In addition, antibodies against glucosylceramide (GlcCer), a glycosphingolipid primarily localized in the cell membrane of *C*. *neoformans* [13], have been shown to inhibit the growth and division of *C*. *neoformans* [14]. Another glycolipid, galactosylceramide (GalCer), has been shown to activate the natural killer cells and increase the immune response induced by malaria vaccines [15]. Despite the evidence on the immunomodulatory properties of glycolipids, they have never been used as a vaccination strategy against *Cryptococcus* infections.

In this study, we investigated the use of glycolipids as a vaccine against cryptococcosis in a mouse model of the disease. Since GalCer has been reported to induce hepatic injury, we focused our efforts on GlcCer, which has also been shown to induce an immune response when administered to mice [16]. We hypothesized that GlcCer will provide a suitable vaccine candidate as this lipid is a major virulence factor of *C*. *neoformans* [17] and anti-GlcCer antibodies inhibit cryptococcal growth and cell division [14]. Administration of GlcCer as a vaccination strategy, the hepatic toxicity of this lipid, and the ability of GlcCer to elicit antibodies depending on the route of administration were investigated in the current study.

## Materials and Methods

### Materials

*Cryptococcus neoformans* (H99) strain was a generous gift from Dr. John Perfect at Duke University Hospital (Durham, NC). GlcCer purified from *Candida utilis* was a gift from Kohjin Life Sciences (Tokyo, Japan). Yeast Peptone Dextrose (YPD) and Yeast Nitrogen Base (YNB) were from BD Technologies (Durham, NC). GlcCer monoclonal antibody IgM clone B11 was generated in the Dr. Del Poeta’s Laboratory. Anti-mouse secondary antibody against IgM conjugated with horse-radish peroxidase was from Santa Cruz Biotechnology (Santa Cruz, CA). Lipopolysaccharide, interferon-γ and Freund’s adjuvant were purchased from Sigma-Aldrich (St. Louis, MO).

### Lipid Analysis

GlcCer purified from *C*. *utilis* was analyzed by electrospray ionization tandem mass spectrometry (ESI-MS/MS) using TSQ Quantum Ultra™ Triple Quadrupole Mass Spectrometer (Thermo Scientific, Waltham, MA). Samples were suspended in a buffer containing 1 mM ammonium formate + 0.2% formic acid in methanol. Samples were delivered to the MS by using direct syringe loop injection at the rate of 10 μl.min^-1^, and were analyzed as [M + H]^+^ in the positive ion mode. A source voltage of 4.5 kV and a collision energy of 20 V was used for experiments. Spectra were recorded for m/z between 200 and 1000. The MS/MS profiles of the abundant peaks were generated using two different collision energies, 20 and 45 V. GlcCer species with 4,8-sphingadienine (d18:2) and 9-methyl-4,9-sphingadienine (d19:2) sphingoid base were detected using parent ion scanning for the fragments of 262.2 and 276.2, respectively. These fragments resulted from the cleavage of amide linkage and subsequent dehydration as described previously [18].

#### Animal Studies

A day prior to infection, a single colony of *C*. *neoformans* was transferred from a YPD agar plate to a YPD broth and grown overnight at 30°C on a rotatory shaker at 250 rpm. After 16–20 hours of growth, the cells were washed with sterile phosphate buffered saline and counted. Four weeks old CBA/J female mice (Jackson Laboratory, Bar Harbor, ME) were used for survival studies. Mice were given an anesthetic injection of 95 mg/kg ketamine and 5 mg/kg xylazine and were infected with 5x10^5^ cells/mouse of *C*. *neoformans* by nasal inoculation and were monitored daily afterwards. GlcCer administration to infected mice was performed daily or weekly as described below. The experimental protocols were approved by the institutional animal care and use committee of Stony Brook University.

### GlcCer Administration Protocols

#### Daily administration

Mice were divided into five groups (n = 10). Groups 1 to 4 received an intraperitoneal injection of 20 μg/day of GlcCer dissolved in ethanol, group 5 were used as control and received 1.3% ethanol in PBS. In groups that were treated with GlcCer, two groups (groups 2 and 4) received complete Freund’s adjuvant at the start of the experiment and incomplete Freund’s adjuvant at the start of week 2. At week 2, group 3, 4, and 5 were challenged with 5x10^5^ *C*. *neoformans* cells. Groups 1 and 2 were used as control to monitor the response of animals to GlcCer and GlcCer + incomplete Freund’s adjuvant and were not challenged with fungal cells. At weeks 0, 2, 4, and 12 weeks, blood was drawn from mice to examine the presence of GlcCer antibody (**S1 Fig**). Mice were fed ad-libitum and monitored closely for signs of discomfort and meningitis. Mice showing abnormal gait, lethargy, tremor, more than 15% loss of body weight or inability to reach water or food were sacrificed were sacrificed with CO_2_ inhalation followed by cervical dislocation and the survival was counted from that day. At the end of the survival study, tissue fungal burden were examined in mice that survived the infection. This was performed by sacrificing the mice and extracting the organs, and homogenizing in 10 mL sterile PBS using a homogenizer (Stomacher80, Cole-Parmer, Vernon Hills, IL). Organ homogenates were serially diluted 1:10 in PBS and 100 μL was plated on YPD agar plates and incubated at 30°C for 72 hours for CFU count. For histopathology, extracted organs were fixed in 10% formalin before paraffin sectioning and staining with either hematoxylin & eosin or mucicarmine. Images were taken at 40X in a Zeiss Axio Observer microscope (Thornwood, NY) in the brightfield mode.

#### Weekly administration

Mice were divided into three groups (n = 10). On day 1, the mice in group 1 were injected intraperitoneally with 50 μg of GlcCer in the presence of complete Freund’s adjuvant 1:1 (v/v), the mice in group 2 were given an intraperitoneal injection with complete Freund’s adjuvant plus 1.3% ethanol in PBS 1:1 (v/v), and group 3 received 1.3% ethanol in PBS. On day 7, group 1 received an intraperitoneal injection of with 50 μg of GlcCer in the presence of incomplete Freund’s adjuvant, the same treatment was repeated on days 14 and 28. Group 2 received incomplete Freund’s adjuvant on day 7 followed by the same treatment on days 14 and 28. Group 3 received 1.3% ethanol in PBS on day 7 followed by the same treatment on days 14 and 28. All groups of mice were challenged with 5x10^5^ *C*. *neoformans* cells intranasally on day 14. At 0, 14, 28, 42 and 84 days, blood was drawn to check the presence of antibody against GlcCer (**S2 Fig**).

#### Administration in different strains of mice

Mice were divided into six groups (n = 6), groups 1, 2, and 5 included CBA/J mice and groups 3, 4, and 6 included BALB/c mice. On day 1, mice of groups 1–4 were injected with 50 μg of GlcCer in the presence of complete Freund’s adjuvant; intraperitoneal injection was used in groups 1 and 3 and subcutaneous injection was used in groups 2 and 4. After the initial treatment, the mice in groups 1–4 were injected with 50 μg of GlcCer in the presence of incomplete Freund’s adjuvant, which was repeated weekly until week 3. The mice in Groups 5 and 6 were used as control and were injected with 1.3% ethanol in PBS (**S3 Fig**). Blood was drawn from all mice groups every two weeks.

### Toxicity Study

The presence of endotoxins in the purified GlcCer was tested using the E-TOXATE kit (SigmaAldrich, St. Louis, MO), which is intended for detection and semi-quantitation of endotoxins. All experiments were performed according to the instructions provided by the manufacturer. Toxicity studies in animals were performed using 4 weeks old CBA/J female mice. Animals were divided into 5 groups (n = 3). Groups 1 and 3 received an intraperitoneal (i.p.) injection of 20 μg GlcCer per mouse/day, groups 2 and 4 received the same amount of GlcCer in presence of incomplete Freund’s adjuvant. Groups 3 and 5 were challenged with 5x10^5^ cryptococcal cells. At 60 days, blood samples were collected in two tubes: one with K_2_EDTA and the other without K_2_EDTA to allow blood clotting. The blood clot was then centrifuged at 1500 rpm for 10 min, serum was collected and analyzed for liver and kidney blood tests. The non-coagulated blood was used for hematocrit and total blood cells analysis. These tests were performed using MASCOT^TM^ HEMAVET 950FS (Drew Scientific Group, Dusseldorf, Germany).

### Enzyme Linked Immunosorbant Assay (ELISA)

ELISA was performed in a 96-well microtiter plate (Maxisorp NUNC, Sigma Aldrich). First, the wells were coated with 50 μL/well of 160 μg/mL of *C*. *utilis* GlcCer and incubated overnight at 4°C. The plate was then blocked for 30 min with 5% BSA in phosphate buffered saline containing 0.1% Tween 20 (PBST). Serum samples were diluted 1:24 in 1% BSA/PBST and 50 μL were added per well and incubated for 16 hours at 4°C. As positive control, and anti-GlcCer IgM antibody was used (F09) (data not shown). This IgM produces an OD of 0.5 at 450 nm when 50 microliters of 1:64 dilution of 1 mg/ml is used in the ELISA [13]. As negative controls, the secondary IgM (or IgG) antibodies were used alone. After five washes with PBST, the plate was incubated with 50 μL per well of either goat anti-human IgM-HRP diluted 1:50,000 with 1% BSA/PBST or goat anti-mouse secondary IgM diluted 1:30,000 with 1% BSA/PBST, for 2 hours at room temperature. After five washes with PBST, the color was developed with 50 μL/well of tetramethylbenzidine (TMB). The reaction was then stopped with 50 μL of 2 M H_2_SO_4_ and the plate read at 450 nm using FliterMax multiplate reader (Molecular devices. Sunnyvale, CA USA).

### Cytokine Analysis

Cytokine analysis was performed on liver mononuclear cells isolated from 7 weeks old C57BL/6 mice. For these experiments 10^6^ cells/mL were cultured in RPMI1640 medium (Sigma-Aldrich) supplemented with 10% FCS (BioWest, Nuaillé, France), 100 U/ml penicillin G, 100 μg/ml streptomycin and 50 μM 2-mercaptoethanol (Sigma-Aldrich). GlcCer from *C*. *utilis* (100 ng/mL, 1000 ng/mL, and 10000 ng/mL) and α-GalCer (100 ng/mL), dissolved in dimethyl sulfoxide (DMSO), were added to the medium. After 48 hours of incubation the medium was tested for the levels of interferon-γ (IFN-γ), tumor necrosis factor-α (TNF-α), interleukin-4 (IL-4), interleukin-6 (IL-6), interleukin-1β (IL-1β), and interleukin-12 subunit p40 (IL-12p40) using ELISA. All antibodies for ELISA were purchased from Biolegend (San Diego, CA, USA).

#### Statistics

All data are expressed as mean ± standard deviation. No samples or animals were excluded from the analysis. For animal studies, group sizes were chosen when sufficient to reach a statistical power of at least 80% (http://www.statisticalsolutions.net/pss_calc.php). Mice were assigned randomly to treatment groups. Statistical analysis for survival studies was performed using Student-Newman-Keuls *t* test for multiple comparisons using INSTAT or by Kruskal-Wallis test. Statistical analysis for tissue burden was performed using the analysis of variance (ANOVA). Additional statistics were performed using unpaired *t* tests (comparison of two groups) or one-way ANOVA (comparison of three or more groups). Statistical tests were carried out using GraphPad Prism (La Jolla, CA, USA) v. 400 Software for Mac. Replicates used were biological replicates. Results were considered significant at *P* ≤ 0.05.

#### Study approval

The mouse experiments were performed in full compliance with a protocol approved by Stony Brook University (Study #341888–7; IACUC#: 2012–1967) and in compliance with the United States Animal Welfare Act (Public Law 98–198). The experiments were carried out in facilities accredited by the Association for Assessment and Accreditation of Laboratory Animal Care.

## Results

### Characterization of GlcCer Purified from *C*. *utilis*

**Fig 1:**
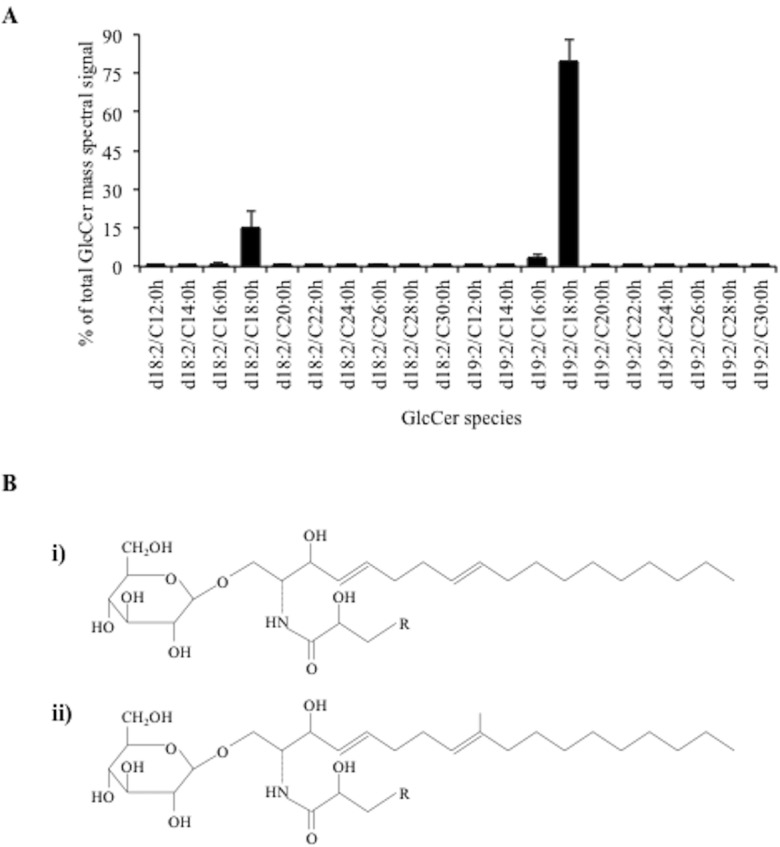
Structure and composition of the analyzed GlcCer’s of *Candida utilis*. (A) Structure of GlcCer: i) GlcCer with 4,8-Sphingadienine (d18:2) sphingoid base. ii) GlcCer with 9-Methyl-4,9-Sphingadienine (d19:2) sphingoid base. The additional carbon chain, R = C9 –C27. (B) Composition analysis of GlcCer as analyzed by ESI-MS. The values are Mean ± SEM and n = 3, where ‘n’ represents analysis from 3 independent purifications. Complete list of data points in presented in **S1 Table**.

The molecular structure and composition of GlcCer purified from *C*. *utilis* was determined using the ESI-MS/MS profiling. MS analysis showed that d18:2 and d19:2 are the predominant sphingoid bases in the structure of GlcCer of *C*. *utilis* (**Fig 1A**), which was in agreement with previous studies [18–20]. The chemical structure of GlcCer species is presented in **Fig 1B**. The most abundant GlcCer species were composed of hydroxylated 16 or 18 carbon fatty acids with GlcCer (d19:2/C18:0h) accounting for about 80% of all GlcCer. The complete list, chemical formula, and molar mass of all detected species is presented in **S1 Table**. The Purified GlcCer was also tested for the presence of endotoxins using the E-TOXATE^TM^ kit from SigmaAldrich. Experiments with GlcCer concentrations of up to 1 g/L did not reveal the presence of endotoxins in the purified GlcCer samples.

### GlcCer Administration Provides Protection against Cryptococcosis in the Mouse Model

**Fig 2:**
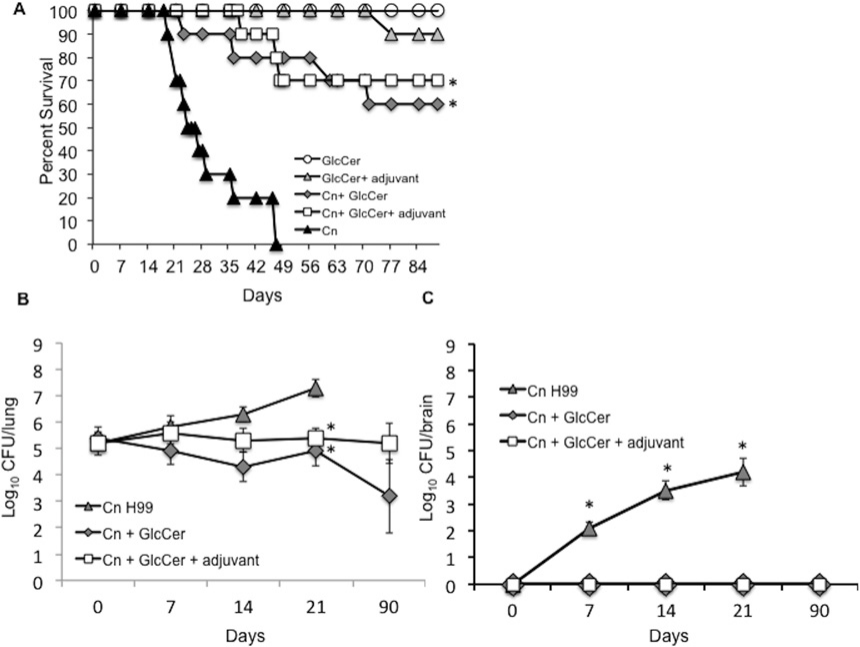
GlcCer administration results in partial immunity against cryptococcosis in mouse models of the disease. (A) Survival of CBA/J mice treated with 20 μg/day of GlcCer or GlcCer + FA and infected intranasally with 5x10^5^ *C*. *neoformans* (Cn) cells. The GlcCer and GlcCer + iFA control groups received treatment, but were not infected. The Cn group were infected, but did not receive treatment. Ten mice per group were used. *, *P<0*.*05* Cn + GlcCer or Cn + GlcCer + adjuvant versus Cn. (B) Fungal tissue burden in the lungs of the mice treated with 20 μg/day of GlcCer or GlcCer + adjuvant and infected intranasally with 5x10^5^ Cn. Three mice per group per day were used. *, *P<0*.*05*, Cn + GlcCer or Cn + GlcCer + adjuvant versus Cn H99 (C). Fungal tissue burden in the brains of the mice treated with 20 μg/day of GlcCer or GlcCer + adjuvant and infected intranasally with 5x10^5^ Cn. Three mice per group per day were used. Day = 0 represents the first day of infection. *, *P<0*.*05*, Cn + GlcCer or Cn + GlcCer = adjuvant versus Cn H99.

GlcCer treatment alone or along with Complete Freund’s Adjuvant (cFA) followed by incomplete Freund’s Adjuvant (iFA) was used as a vaccination strategy against cryptococcosis. For these studies, mice were treated daily with GlcCer with or without adjuvant. Mice treated with 1.3% ethanol in PBS were used as control. After two weeks, mice were infected with 5x10^5^ *C*. *neoformans* cells intranasally and monitored for survival. It was observed that 60% of the mice that had received GlcCer and 70% of the mice that had received GlcCer and adjuvant survived the infection during the course of the observation (90 days post-infection). In contrast, all the mice that had received PBS (control group) succumbed to the infection in an average of 25 days (**Fig 2A**). Intraperitoneal injection of GlcCer alone or GlcCer in the absence of *C*. *neoformans* did not result in mouse mortality. One mouse receiving GlcCer along with iFA died unexpectedly. It is unlikely that this was due to toxic effect of GlcCer + iFA trearment as it did not happen in subsequent experiments.

Fungal tissue burden was assessed in the lungs and brain of infected mice vs. mice that received GlcCer and GlcCer and adjuvant (**Fig 2B** and **2C**). The number of fungal cells in the lungs of the infected group that received GlcCer and GlcCer and adjuvant remained steady at about Log_10_ CFU of 5 throughout the experiment. However, in infected mice that received no treatment a steady increase in the number of fungal cells was observed. At 21 days post-infection the number of fungal cells increased to Log_10_ CFU of 7.3 ± 0.3, almost 100 times more than the treated groups (**Fig 2B**). Dissemination of fungal cells to the brain was observed as early as 7 days post-infection in the infected group that received no treatment and increased steadily until 21 days after the infection. However, no fungal cells were found in the brains of the infected group that received GlcCer and GlcCer and adjuvant (**Fig 2C**). It is worth noting that all three mice showed fungal cells in their liver and kidney in the treated groups (data not shown), suggesting that the mortality observed in these groups might have been caused by infections in organs other than the brain.

**Fig 3:**
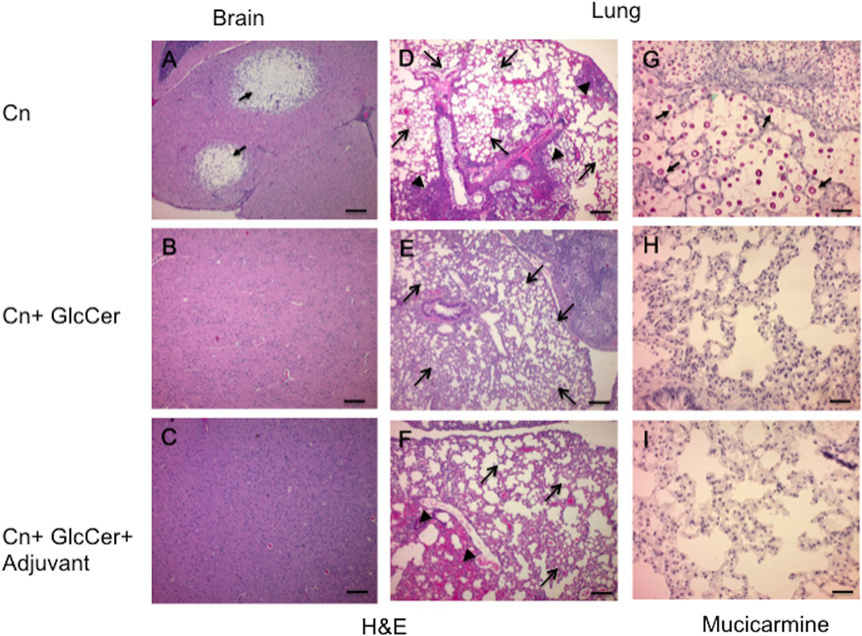
Histopathology of lungs and brain of mice infected with *C*. *neoformans* and treated with GlcCer vs. untreated mice. A) Brain of untreated mice stained with H&E. Colonization of *C*. *neoformans* (Cn) cells in the brain is shown with arrows. Histology was performed 20 days post-infection. B & C) Brain of infected mice treated with GlcCer or GlcCer + FA. Histology was performed 20 days post-infection. Images showed no abnormality in the brain tissue and no *C*. *neoformans* cells was found, confirming CFU data illustrated in **Fig 2C**, **2D** and **2G**) Lungs of untreated mice stained with H&E or mucicarmine. Histology was performed 20 days post-infection. Significant lung inflammation is present (arrowheads in D), where the lung tissue is infiltrated with numerous macrophages, lymphocytes and neutrophils. *C*. *neoformans* cells are readily visible in these areas (not shown) and in the alveoli (arrows in G). E & H) Lungs of mice treated with GlcCer and stained with H&E or mucicarmine. Histology was performed 90 days post-infection. Arrows in E illustrate normal lung structure. F & I) Lung of mice treated with GlcCer + adjuvant and stained with H&E or mucicarmine. Histology was performed 90 days post-infection. Arrows in F illustrate normal lung structure. Arrowheads in F illustrate some lung inflammation with no *C*. *neoformans* cells present at this particular site of inflammation (I), although they were present in other fields. Three mice per group were used in all histology experiments. The images shown are representative fields of the entire organ. Black bar in A, B an C, 200 μm; black bar in D, E and F, 200 μm; black bar in G, H and I, 40 μm.

Histopathological tissue analysis closely agreed with analysis of the tissue burden. Haematoxyline & eosin (H&E) staining revealed severe colonization (black arrows) of the brain by fungal cells in the control group that had received PBS prior to infection (**Fig 3A**). In contrast, the brain tissue from mice vaccinated with either GlcCer alone (**Fig 3B**) and GlcCer along with adjuvant (**Fig 3C**) showed no cryptococcal cells and no major abnormality in the brain structure. Importantly, vaccination with GlcCer or GlcCer + adjuvant resulted in normal lung structure (**Fig** 3B and 3C). Massive infiltrations of the lungs by *C*. *neoformans* cells were observed in the control group (**Fig** 3G) with significant infiltration by macrophages, lymphocytes and neutrophils, which subverted the normal structure of the lung (**Fig** 3D). In contrast, minimal subversion of the lung structure was observed in the groups treated with GlcCer and GlcCer and adjuvant (**Fig 3E** and **3F**). Mucicarmine staining revealed the presence of fungal cells (dark purple) in the lungs of the control group (**Fig 3G**) and not in the lungs of the groups treated with GlcCer and GlcCer and complete Freund’s adjuvant followed by incomplete Freund’s adjuvant (**Fig 3H** and **3I**). Detailed analysis of fungal burden for each organ is presented with CFU analysis in **Fig 2B** and **2C**.

### Mice Tolerate Daily Administration of GlcCer

**Fig 4:**
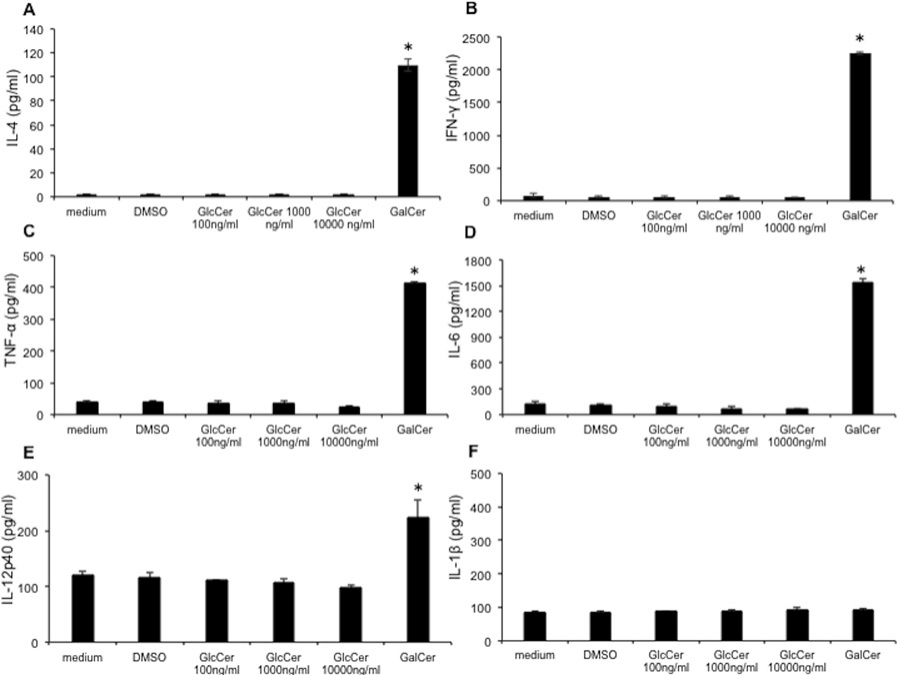
Detection of cytokines in the medium of mice liver mononuclear cells following glycolipid treatment. Detection of A) IL-4, B) IFN-ϒ. C) TNF-α, D) IL-6, E) IL-12p40, and F) IL-1β. Plots are the result of ELISA experiments performed on the media of cells after 48 hours of incubation with various concentration of β-GlcCer or 100 ng/ml of α-GalCer. * *P < 0*.*05*, GalCer versus medium, DMSO or GlcCer groups.

While other sphingolipids have been shown to be potent immunostimulators, their use has been hampered by concerns over their toxicity. A prime example in this regard is α-galactosyl ceramide (α-GalCer), an immunostimulating sphingolipid that has been used as an adjuvant for a variety of vaccines [15, 21–23], but has been shown to exert cytokine-mediated hepatic injury [24]. To examine whether such a response might be observed after the injection of GlcCer liver mononuclear cells were isolated from C57BL/6 mice and were incubated with various amounts of GlcCer (100 ng/mL to 10000 ng/mL) for 48 hours and were tested for the production of IFN-γ, IL-4, TNF-α, IL-6, IL-1β, and IL-12p40 using ELISA. Several of these cytokines have been reported to increase following α-GalCer administration in mice [24]. For these studies, 100 ng/mL of α-GalCer was used as a positive and DMSO was used as a negative control. While various amounts of GlcCer did not cause an increase in the amount of cytokines, a significant increase in the amounts of IFN-γ, IL-4, TNF-α, IL-6, and IL-12p40 was observed after incubation with α-GalCer (**Fig 4**). These results suggested the lack of liver toxicity after the administration of GlcCer in mice. This possibility was further investigated by collecting blood from healthy, infected, and GlcCer and FA treated mice and performing liver functions tests. GlcCer administration did not increase the levels of alkaline phosphatase (ALP), alanine aminotransferase (ALT), and total bilirubin (TBILI); an increase in the level of aspartate aminotransferase (AST) was observed, but the average value of AST remained in the normal range (**S2 Table**). Finally, total leukocytes and erythrocytes were tested following GlcCer administration and did not show an increase above the normal ranges (**S3** and **S4 Tables)**. Taken together, these data suggest that GlcCer does not induce toxicity in mice following 90 days of daily administration.

### Administration of GlcCer Elicits a Modest Antibody Response

**Fig 5:**
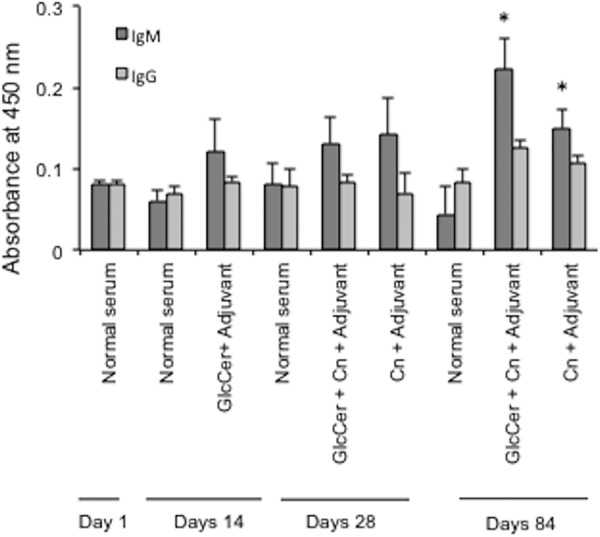
Detection of antibody against GlcCer in mice sera. IgM and IgG detection in the sera of CBA/J mice treated with weekly administration of 50 μg GlcCer and complete Freund’s adjuvant (cFA) followed by iFA after a week. Plot shows the results of ELISA experiments performed using purified GlcCer as antigen. Data represents analysis from 3 independent experiments. *, *P<* 0.05, IgM GlcCer + Cn + Adjuvant at day 84 versus normal serum IgM at Day 84.

In an effort to understand the mechanisms of GlcCer-induced immunity to *Cryptococcus* infection, the production of anti-GlcCer antibody in mice following GlcCer administration was examined. Anti-GlcCer antibody has been shown to inhibit the growth and budding of *C*. *neoformans* cells [14]. Our initial studies revealed that daily administration with 20 μg of GlcCer and adjuvant and weekly administration with 50 μg of GlcCer and cFA followed by weekly administration of GlcCer + iFA in the following weeks result in similar amount of IgM production (data not shown). Thus, to reduce the amount of injections, the latter treatment regime was used for antibody response experiments. To this aim, mice were pretreated with cFA, and GlcCer + cFA for one week and then with GlcCer + iFA weekly in the following weeks until week 3. Two weeks after the start of treatment, the mice were infected with a lethal dose of *C*. *neoformans*. Serum from uninfected mice and mice infected and treated with adjuvant, and GlcCer + adjuvant were examined for anti-GlcCer antibody production at different times post-infection (**Fig 5**). Injection of GlcCer + adjuvant led to a significant, but modest increase in IgM production even prior to infection with *C*. *neoformans* (0.121 ± 0.039 compared to 0.061 ± 0.014 a.u. of absorbance at 450 nm in control). At two weeks post-infection (day 28) the antibody titers remained significantly higher than the uninfected mice, but no statistical significant difference compared to infected mice was observed. At 14 and 70 days post-infection (the last day of observation), no significant changes in IgG levels were observed and they remained similar to the values observed at the start of infection. However, IgM levels showed a statistical significant increase over time with their highest values observed at 70 days post-infection (day 84). Interestingly, the increase in IgM levels was only observed in mice pretreated with GlcCer + adjuvant suggesting that GlcCer played an important role in IgM production.

**Fig 6:**
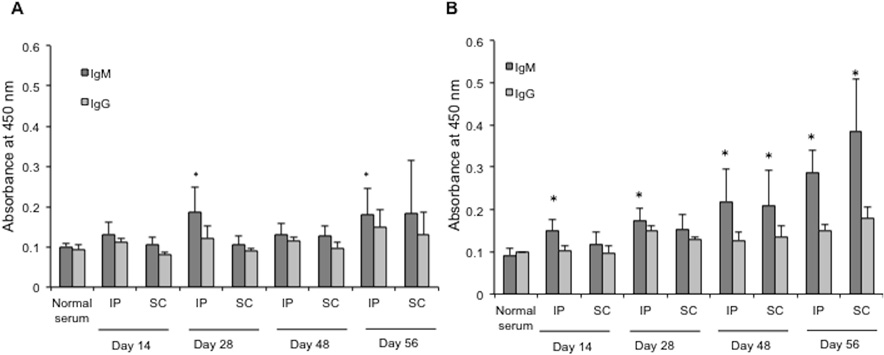
Detection of GlcCer antibody in the sera of different strains of mice following different route of administration. A) Sera of CBA/J mice treated with weekly administration of GlcCer + adjuvant using interaperitoneal (IP) or subcutaneous (SC) administration. *, *P* < 0.05, IgM day 28 or day 56 versus IgM normal serum (day 1). B) Sera of BALB/c mice treated with weekly administeration of GlcCer + FA. Six mice per group were used. Plots show the results of ELISA experiments performed using purified GlcCer as antigen. *, *P* < 0.05, IgM at day 14, 28, 48, or 56 versus IgM normal serum.

The induction of anti-GlcCer IgM antibody by GlcCer + adjuvant was not limited to a certain mouse strain. Treatment of CBA/J mice or BALB/C mice with GlcCer + Freund’s adjuvant in the absence of infection led to an increase in antibody production compared to uninfected mice (**Fig 6A** and **6B**). A modest and not significant increase in IgG was observed and increased antibody titer was primarily limited to IgM. While the trends suggested increased IgM production in BALB/c compared to the CBA/J mice, no significant difference in IgM levels between mice strains was observed at any of the time points. This observation revealed that while GlcCer + adjuvant was able to stimulate antibody production in the BALB/c mouse model and antibody production was not superior compared to the CBA/J model. In light of this observation, survival studies were not performed in the BALB/c mouse model. Intraperitoneal and subcutaneous injection of GlcCer + Freund’s adjuvant both resulted in increased IgM production and no significant difference was generally observed depending on the route of administration (except for day 28 in CBA/J mice).

## Discussion

In this study, we investigated the possibility of using GlcCer isolated from a non-pathogenic fungus (*Candida utilis*) as a vaccination strategy against *C*. *neoformans* infections. Our results demonstrate that purified GlcCer effectively protects mice against a lethal intranasal challenge of *C*. *neoformans*. GlcCer administration prevented the dissemination of *C*. *neoformans* cells to the brain and did not induce hepatic injury in mice. Antibody production against GlcCer was observed in mice and was independent of the route of administration and the strains of mice. These results suggest that GlcCer can be a suitable candidate as a vaccine against cryptococcosis.

Vaccination with GlcCer and GlcCer with Freund’s adjuvant resulted in 60% to 70% mouse survival (**Fig 2**). To the best of our knowledge, a complete (100%) survival has only been observed with live-attenuated *Cryptococcus* strains [10–12]. Studies in which other vaccination strategies have been used have generally reported lower survival rates. This has been the case for GXM-TT vaccines [4], galactoxylomannan-BSA conjugates [25], mimotopes of cryptococcal capsular polysaccharide [6, 7], *C*. *neoformans* culture filtrate + complete FA [8], and extracellular polysaccharide deacetylases [26]. Fungal burden (**Fig 2B** and **2C**) and histopathology studies (**Fig 3**), suggest that fungal cells did not penetrate the brain in mice treated with GlcCer and GlcCer + adjuvant. Of particular importance was the fact that GlcCer did not stimulate hepatic injury, which increases its potential for clinical use. This was corroborated by liver function assays and measurement of cytokines generated from mouse liver mononuclear cells. This is significant as α-GalCer, which has been used alongside vaccines to stimulate the immune response [15, 21–23], results in significant hepatic injury [24, 27, 28]. Administration of α-GalCer has been reported to increase TNF-α, IFN-γ, IL-2, IL-4, and IL-6 production and lead to a hepatic injury that is mediated by TNF-α [24]. We observed a similar increase in TNF-α, IFN-γ, IL-4, IL-6 production by liver mononuclear cells upon the addition of 100 ng/mL of α-GalCer, but no increase in the level of these cytokines was observed after the addition of GlcCer at 100 times higher concentration (**Fig 4**).

Administration of GlcCer resulted in anti-GlcCer antibody production. Mechanistic studies have revealed that GlcCer is involved in the regulation of fungal cell replication in environments characterized by neutral/alkaline environment [13, 17, 29]. *C*. *neoformans* mutants that lack GlcCer cannot progress through the cell cycle in neutral/alkaline pH and lose their ability to establish virulence in the mouse model [17, 30, 31]. Antibodies generated against GlcCer purified from *C*. *neoformans* have been shown to be fungistatic against *C*. *neoformans* and inhibit cell growth and budding [14]. Our results reveal a time-dependent increase in both IgM and IgG production upon GlcCer administration (**Fig 5**), which might contribute to fungal growth inhibition. However, the amount of antibody production following treatment with GlcCer or GlcCer + adjuvant does not seem high enough to be the only mechanism of protection. This notion is further supported by the fact that only a slight increase in antibody production was observed in the presence of GlcCer and Freund’s adjuvant. Freund’s adjuvant was used as the adjuvant of choice in this study due to its ability to effectively stimulate antibody production against a wide range of antigenic molecules. The slight increase in antibody production during co-treatment with GlcCer and adjuvant suggests that an antibody-mediated protection is unlikely to be the only mechanism of protection. Alternative mechanisms, such as the activation of natural killer cells upon GlcCer administration [16], will be further investigated. Also, in this study we did not try less toxic adjuvants [32], but we are planning to do so in the near future. This is important as Freund’s adjuvant is not recommended for human use.

Antibody production was not dependent on the strains of mice and the route of administration. While slight differences in IgM production were observed between CBA/J and BALB/c mice, both strains responded to GlcCer. The observation that there was no difference in antibody production between the intraperitoneal and subcutaneous injection was particularly significant. Intraperitoneal injection, while common in studies with rodents, is rarely used for vaccine administration in humans. Thus, the possibility of subcutaneous administration adds to the clinical potential of the vaccine. In summary, our results suggest that GlcCer can be a potential vaccine candidate against cryptococcosis, GlcCer administration elicits an antibody response without causing hepatic injury. These results are of potential clinical significance as there is currently a need for the development and improvement of fungal vaccines.

## Supporting Information

S1 Fig Schematic representation of daily glucosylceramide administration to mice. Treatments with controls (PBS and ethanol) are not shown for clarity. For experimental details please refer to Materials and Methods. cFA = complete Freund’s adjuvant; iFA = incomplete Freund’s adjuvant.
(TIFF)

S2 Fig Schematic representation of weekly glucosylceramide administration to mice. Treatments with controls (PBS and ethanol) are not shown for clarity. For experimental details please refer to Materials and Methods.
(TIFF)

S3 Fig Schematic representation of the weekly glucosylceramide injection using different routes of administration. For experimental details please refer to Materials and Methods.
(TIFF)

S1 Table Analysis of GlcCer species purified from *C*. *utilis* by ESI-MSMS. (DOCX)

S2 Table Results of the liver function assays performed on mice infected and treated with GlcCer and Freund’s adjuvant. Studies were performed on CBA/J mice (three mice per group), GlcCer was administered daily by intraperitoneal injection 20μg/day for 90 days prior to analysis.
(DOCX)

S3 Table Total leukocyte counts in the blood of treated and untreated mice with or without *C*. *neoformans* infection. Studies were performed on CBA/J mice (three mice per group), GlcCer was administered daily by intraperitoneal injection 20μg/day for 90 days prior to analysis.
(DOCX)

S4 Table Total leukocyte counts in the blood of treated and untreated mice with or without *C*. *neoformans* infection. Studies were performed on CBA/J mice (three mice per group), GlcCer was administered daily by intraperitoneal injection 20μg/day for 90 days prior to analysis.
(DOCX)
