# Supplementary figures and images for: Glucosylceramide Administration as a Vaccination Strategy in Mouse Models of Cryptococcosis
Source: PLoS One. 2016 Apr 15;11(4):e0153853. doi: 10.1371/journal.pone.0153853 (PMC4833283; doi:10.1371/journal.pone.0153853)

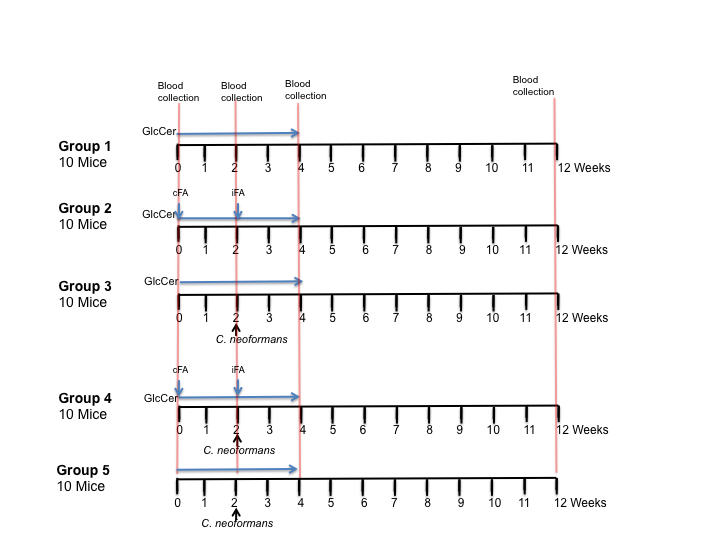

Supplement: S1 Fig — Treatments with controls (PBS and ethanol) are not shown for clarity. For experimental details please refer to Materials and Methods. cFA = complete Freund’s adjuvant; iFA = incomplete Freund’s adjuvant. (TIFF) [file pone.0153853.s001.tiff]

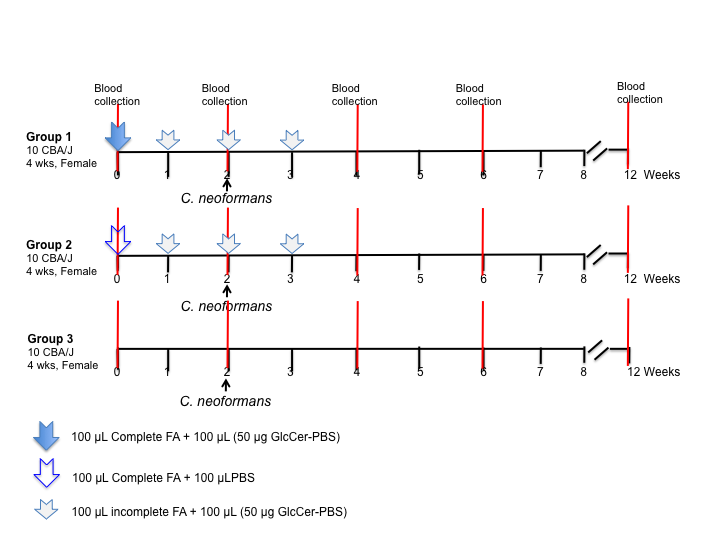

Supplement: S2 Fig — Treatments with controls (PBS and ethanol) are not shown for clarity. For experimental details please refer to Materials and Methods. (TIFF) [file pone.0153853.s002.tiff]

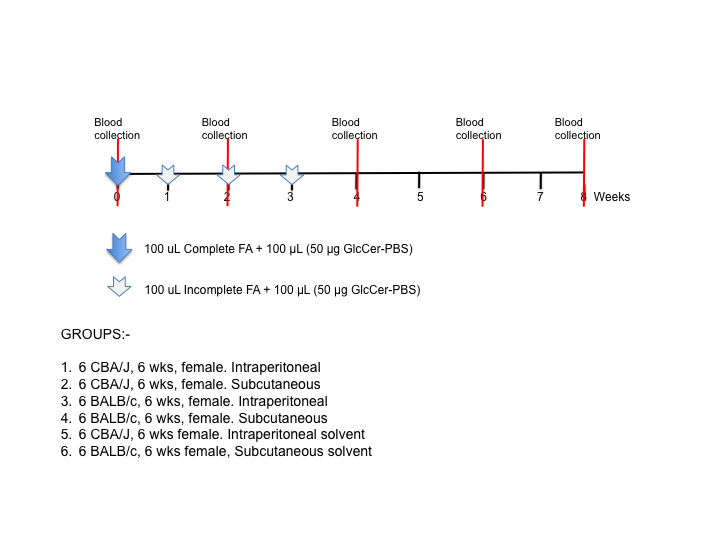

Supplement: S3 Fig — For experimental details please refer to Materials and Methods. (TIFF) [file pone.0153853.s003.tiff]
