# Supplementary material for: Glucosylceramide Administration as a Vaccination Strategy in Mouse Models of Cryptococcosis
Source: PLoS One. 2016 Apr 15;11(4):e0153853. doi: 10.1371/journal.pone.0153853 (PMC4833283; doi:10.1371/journal.pone.0153853)
Supplement: S1 Table — (DOCX) [file pone.0153853.s004.docx]

**S1 Table.** Analysis of GlcCer species purified from *C. utilis* by ESI-MSMS.

| **S. No** | **GlcCer species** | **Formula** | **Exact Molar mass** | **Sphingoid base** | **Fatty acid** | **Mean ± SEM** | **Systemic Nomenclature** |
| --- | --- | --- | --- | --- | --- | --- | --- |
| 1 | d18:2/C12:0h | C36H67NO9 | 657.48 | 4,8-sphingadienine (d18:2) | C12:0h | 0.000 ± 0.000 | N-2'-hydroxydodecanoyl-l-O-β-D-glucopyranosyl-4,8-sphingadienine |
| 2 | d18:2/C14:0h | C38H71NO9 | 685.51 | 4,8-sphingadienine (d18:2) | C14:0h | 0.000 ± 0.000 | N-2'-hydroxytetradecanoyl-l-O-β-D-glucopyranosyl-4,8-sphingadienine |
| 3 | d18:2/C16:0h | C40H75NO9 | 713.54 | 4,8-sphingadienine (d18:2) | C16:0h | 0.807 ± 0.702 | N-2'-hydroxyhexadecanoyl-l-O-β-D-glucopyranosyl-4,8-sphingadienine |
| 4 | d18:2/C18:0h | C42H79NO9 | 741.57 | 4,8-sphingadienine (d18:2) | C18:0h | 14.779 ± 7.081 | N-2'-hydroxyoctadecanoyl-l-O-β-D-glucopyranosyl-4,8-sphingadienine |
| 5 | d18:2/C20:0h | C44H83NO9 | 769.60 | 4,8-sphingadienine (d18:2) | C20:0h | 0.480 ± 0.588 | N-2′-hydroxyeicosanoyl-l-O-β-D-glucopyranosyl-4,8-sphingadienine |
| 6 | d18:2/C22:0h | C46H87NO9 | 797.64 | 4,8-sphingadienine (d18:2) | C22:0h | 0.000 ± 0.000 | N-2′-hydroxydocosanoyl-l-O-β-D-glucopyranosyl-4,8-sphingadienine |
| 7 | d18:2/C24:0h | C48H91NO9 | 825.67 | 4,8-sphingadienine (d18:2) | C24:0h | 0.000 ± 0.000 | N-2′-hydroxytetracosanoyl-l-O-β-D-glucopyranosyl-4,8-sphingadienine |
| 8 | d18:2/C26:0h | C50H95NO9 | 853.70 | 4,8-sphingadienine (d18:2) | C26:0h | 0.324 ± 0.259 | N-2′-hydroxyhexacosanoyl-l-O-β-D-glucopyranosyl-4,8-sphingadienine |
| 9 | d18:2/C28:0h | C52H99NO9 | 881.73 | 4,8-sphingadienine (d18:2) | C28:0h | 0.000 ± 0.000 | N-2′-hydroxyoctacosanoyl-l-O-β-D-glucopyranosyl-4,8-sphingadienine |
| 10 | d18:2/C30:0h | C54H103NO9 | 909.76 | 4,8-sphingadienine (d18:2) | C30:0h | 0.249 ± 0.304 | N-2′-hydroxytricontanoyl-l-O-β-D-glucopyranosyl-4,8-sphingadienine |
| 11 | d19:2/C12:0h | C37H69NO9 | 671.50 | 9-methyl-4,9-sphingadienine (d19:2) | C12:0h | 0.000 ± 0.000 | N-2'-hydroxydodecanoyl-l-O-β-D-glucopyranosyl-9-methyl-4,8-sphingadienine |
| 12 | d19:2/C14:0h | C39H73NO9 | 699.53 | 9-methyl-4,9-sphingadienine (d19:2) | C14:0h | 0.000 ± 0.000 | N-2'-hydroxytetradecanoyl-l-O-β-D-glucopyranosyl-9-methyl-4,8-sphingadienine |
| 13 | d19:2/C16:0h | C41H77NO9 | 727.56 | 9-methyl-4,9-sphingadienine (d19:2) | C16:0h | 3.419 ± 1.450 | N-2'-hydroxyhexadecanoyl-l-O-β-D-glucopyranosyl-9-methyl-4,8-sphingadienine |
| 14 | d19:2/C18:0h | C43H81NO9 | 755.59 | 9-methyl-4,9-sphingadienine (d19:2) | C18:0h | 79.880 ± 8.407 | N-2'-hydroxyoctadecanoyl-l-O-β-D-glucopyranosyl-9-methyl-4,8-sphingadienine |
| 15 | d19:2/C20:0h | C45H85NO9 | 783.62 | 9-methyl-4,9-sphingadienine (d19:2) | C20:0h | 0.061 ± 0.075 | N-2′-hydroxyeicosanoyl-l-O-β-D-glucopyranosyl-9-methyl-4,8-sphingadienine |
| 16 | d19:2/C22:0h | C47H89NO9 | 811.65 | 9-methyl-4,9-sphingadienine (d19:2) | C22:0h | 0.000 ± 0.000 | N-2′-hydroxydocosanoyl-l-O-β-D-glucopyranosyl-9-methyl-4,8-sphingadienine |
| 17 | d19:2/C24:0h | C49H93NO9 | 839.68 | 9-methyl-4,9-sphingadienine (d19:2) | C24:0h | 0.000 ± 0.000 | N-2′-hydroxytetracosanoyl-l-O-β-D-glucopyranosyl-9-methyl-4,8-sphingadienine |
| 18 | d19:2/C26:0h | C51H97NO9 | 867.71 | 9-methyl-4,9-sphingadienine (d19:2) | C26:0h | 0.000 ± 0.000 | N-2′-hydroxyhexacosanoyl-l-O-β-D-glucopyranosyl-9-methyl-4,8-sphingadienine |
| 19 | d19:2/C28:0h | C53H101NO9 | 895.74 | 9-methyl-4,9-sphingadienine (d19:2) | C28:0h | 0.000 ± 0.000 | N-2′-hydroxyoctacosanoyl-l-O-β-D-glucopyranosyl-9-methyl-4,8-sphingadienine |
| 20 | d19:2/C30:0h | C55H105NO9 | 923.78 | 9-methyl-4,9-sphingadienine (d19:2) | C30:0h | 0.000 ± 0.000 | N-2′-hydroxytricontanoyl-l-O-β-D-glucopyranosyl-9-methyl-4,8-sphingadienine |
