# Supplementary material for: Glucosylceramide Administration as a Vaccination Strategy in Mouse Models of Cryptococcosis
Source: PLoS One. 2016 Apr 15;11(4):e0153853. doi: 10.1371/journal.pone.0153853 (PMC4833283; doi:10.1371/journal.pone.0153853)
Supplement: S2 Table — Studies were performed on CBA/J mice (three mice per group), GlcCer was administered daily by intraperitoneal injection 20μg/day for 90 days prior to analysis. (DOCX) [file pone.0153853.s005.docx]

**S2 Table. Results of the liver function assays performed on mice infected and treated with GlcCer and Freund’s adjuvant.** Studies were performed on CBA/J mice (three mice per group), GlcCer was administered daily by intraperitoneal injection 20μg/day for 90 days prior to analysis.

| **Test (Units)** | **Normal range** | **Control**  **(n=3)** | **GlcCer**  **(n=3)** | **GlcCer+ IFA (n=3)** | **Cn+ GlcCer (n=3)** | **Cn+ GlcCer+ IFA (n=3)** |
| --- | --- | --- | --- | --- | --- | --- |
| ALP (U/L) | 35.00-101.00 | 114.33 ± 13.27 | 92.30 ± 12.20 | 77.00 ± 8.80 | 71.00 ± 12.71 | 68.66 ± 8.62 |
| ALT (U/L) | 17.00-32.00 | 28.00 ± 1.73 | 27.30 ± 8.00 | 23.66 ± 2.10 | 37.00 ± 15.71 | 27.33 ± 6.65 |
| AST (U/L) | 54.00-120.00 | 48.00 ± 5.56 | 116.60 ± 61.00 | 78.00 ± 14.90 | 108.66 ± 17.00 | 101.66 ± 17.92 |
| TBILI (mg/dL) | 0.0 – 1.0 | 0.23 ± 0.05 | 0.13 ± 0.05 | 0.13 ± 0.05 | 0.16 ± 0.11 | 0.17 ± 0.12 |

ALP, Alkaline phosphatase; ALT, Alanine aminotransferase; AST, Aspartate aminotransferase; TBILI, Total bilirubin.
