# Supplementary material for: Glucosylceramide Administration as a Vaccination Strategy in Mouse Models of Cryptococcosis
Source: PLoS One. 2016 Apr 15;11(4):e0153853. doi: 10.1371/journal.pone.0153853 (PMC4833283; doi:10.1371/journal.pone.0153853)
Supplement: S3 Table — Studies were performed on CBA/J mice (three mice per group), GlcCer was administered daily by intraperitoneal injection 20μg/day for 90 days prior to analysis. (DOCX) [file pone.0153853.s006.docx]

**S3 Table. Total leukocyte counts in the blood of treated and untreated mice with or without *C. neoformans* infection.** Studies were performed on CBA/J mice (three mice per group), GlcCer was administered daily by intraperitoneal injection 20μg/day for 90 days prior to analysis.

| **Leukocytes (Units)** | **Normal range** | **Control**  **(n=3)** | **GlcCer**  **(n=3)** | **GlcCer+ IFA (n=3)** | **Cn+ GlcCer (n=3)** | **Cn+ GlcCer+ IFA (n=3)** |
| --- | --- | --- | --- | --- | --- | --- |
| WBC (10^3/μL) | 1.80 - 10.70 | 4.11 ± 0.56 | 3.40 ± 0.96 | 5.60 ± 10.00 | 5.10 ± 0.50 | 5.26 ± 0.64 |
| NE (10^3/μL) | 0.10 - 2.40 | 0.40 ± 0.03 | 0.80 ± 0.10 | 0.59 ± 0.90 | 0.90 ± 0.06 | 1.44 ± 0.07 |
| LY (10^3/μL) | 0.90 - 9.30 | 3.34 ± 0.52 | 3.42 ± 0.67 | 3.69 ± 0.45 | 3.38 ± 0.66 | 2.81 ± 0.50 |
| MO (10^3/μL) | 0.00 - 0.40 | 0.32 ± 0.05 | 0.13 ± 0.03 | 0.11 ± 0.02 | 0.09 ± 0.02 | 0.07 ± 0.01 |
| EO (10^3/μL) | 0.00 - 0.20 | 0.02 ± 0.03 | 0.10 ± 0.02 | 0.06 ± 0.00 | 0.09 ± 0.00 | 0.12 ± 0.03 |
| BA (10^3/μL) | 0.00 - 0.20 | 0.01 ± 0.01 | Not detected | 0.01 ± 0.00 | Not detected | Not detected |

WBC, White blood cell; NE, Neutrophiles; LY, lymphocytes; MO, Monocytes; EO, Eosinophiles; BA, Basophiles
