# Supplementary material for: Glucosylceramide Administration as a Vaccination Strategy in Mouse Models of Cryptococcosis
Source: PLoS One. 2016 Apr 15;11(4):e0153853. doi: 10.1371/journal.pone.0153853 (PMC4833283; doi:10.1371/journal.pone.0153853)
Supplement: S4 Table — Studies were performed on CBA/J mice (three mice per group), GlcCer was administered daily by intraperitoneal injection 20μg/day for 90 days prior to analysis. (DOCX) [file pone.0153853.s007.docx]

**S4 Table. Total leukocyte counts in the blood of treated and untreated mice with or without *C. neoformans* infection.** Studies were performed on CBA/J mice (three mice per group), GlcCer was administered daily by intraperitoneal injection 20μg/day for 90 days prior to analysis.

| **Erythrocytes (Units)** | **Normal range** | **Control**  **(n=3)** | **GlcCer**  **(n=3)** | **GlcCer+ IFA (n=3)** | **Cn+ GlcCer (n=3)** | **Cn+ GlcCer+ IFA (n=3)** |
| --- | --- | --- | --- | --- | --- | --- |
| RBC (10^6/μL) | 6.36-9.42 | 7.33 ± 0.24 | 6.93 ± 0.97 | 77.98 ± 0.51 | 88.50 ± 0.56 | 8.10 ± 0.84 |
| Hb (g/dL) | 11.00-15.10 | 11.15 ± 0.07 | 11.80 ± 1.47 | 13.10 ± 1.10 | 13.70 ± 0.56 | 12.26 ± 1.35 |
| MCV (fL) | 45.40-60.30 | 53.67 ± 0.25 | 50.66 ± 6.43 | 447.00 ± 3.46 | 334.66 ± 23.28 | 45.33 ± 1.50 |
| MCH (pg) | 14.10-19.30 | 15.00 ± 0.30 | 17.06 ± 0.80 | 116.43 ± 1.30 | 226.10 ± 16.41 | 15.13 ± 0.23 |
| MCHC (g/dL) | 30.20-34.20 | 28.05 ± 0.45 | 34.06 ± 2.70 | 335.10 ± 0.70 | 228.33 ± 11.30 | 33.53 ± 1.15 |
| **Thrombocytes (Units)** | | | | | | |
| PLT (10^3^/μL) | 592.00-297.00 | 372.33 ± 221.24 | 624.33 ± 245.00 | 8801.60 ± 185.20 | 4493.00 ± 416.00 | 620.00 ± 107.00 |

RBC, Red blood cell; Hb, Hemoglobin; MCV, Mean corpuscular vol.; MCH, Mean corpuscular hemoglobin; MCHC, Mean corpuscular hemoglobin concentration; PLT, Platelets.
